# Comprehensive metabolomic characterization of defense reprogramming in *Castanea mollissima* under *Oligonychus ununguis* feeding stress

**DOI:** 10.1371/journal.pone.0338168

**Published:** 2026-05-15

**Authors:** Xinfang Zhang, Shuhang Zhang, Ying Li, Yan Guo, Jinyu Liu, Jiayi Liu, Liying Fan, Guangpeng Wang

**Affiliations:** Changli Fruit Institute, Hebei Academy of Agriculture and Forestry Sciences, Changli, Hebei, China; Osmania University, INDIA

## Abstract

Piercing-sucking arthropods such as the chestnut red mite (CRM) *Oligonychus ununguis* impair leaf tissues by extracting nutrients from mesophyll cells, thereby lowering the photosynthetic capacity and nut yield in Chinese chestnut (*Castanea mollissima* Bl.). Despite its economic significance, the metabolic mechanisms underlying chestnut responses to mite infestation remain poorly understood. Comparative leaf metabolomic analyses were conducted on four cultivars exhibiting different resistance levels to elucidate these mechanisms. The leaf chlorosis index was used to assess resistance, and three pairwise comparison groups were established accordingly. Secondary metabolomic profiling demonstrated that resistant varieties exhibited more pronounced metabolic reprogramming following infestation than susceptible cultivars. Among the secondary metabolites detected, flavonoids and phenolic acids were predominant. The Kyoto Encyclopedia of Genes and Genomes (KEGG) pathway enrichment analysis identified numerous differentially accumulated metabolites (DAMs) associated with flavonoid, flavonol, and phenylpropanoid biosynthesis pathways. Remarkably, 27 phenylpropanoids and flavonoids were significantly accumulated in resistant cultivars but were largely undetected or showed no substantial variation in vulnerable cultivars. These findings indicate that specific phenylpropanoids and flavonoids, along with their corresponding biosynthetic networks, are integral to the resistance of the species against infestation by chestnut red mites. This study provides a metabolic foundation for breeding mite-resistant chestnut varieties.

## Introduction

Spider mites are globally important pests that substantially reduce yields of agricultural crops and fruit trees, resulting in significant economic losses [1]. Intensive and prolonged use of pesticides has accelerated their evolutionary adaptation, leading to resistance to multiple classes of chemical acaricides [2,3]. Consequently, enhancing host plant resistance has become a primary objective in fruit tree breeding programs aimed at advancing sustainable agriculture systems [4].

Chinese chestnut (*Castanea mollissima* Bl.), an ancient domesticated member of the Fagaceae family, was first cultivated in China, which continues to be the world’s leading chestnut producer. This species is prized for its excellent nut quality and remarkable resilience to various environmental stresses [5]. The chestnut red mite (*Oligonychus ununguis*) represents a significant pest in northern cultivation regions, such as Shandong, Hebei, and Beijing. The damage it causes leads to chlorosis along the leaf veins, which severely impairs photosynthesis and lowers nut yield and quality in both the current and the following season [6]. These impacts result in considerable financial losses for growers, with mite management accounting for approximately 20–25% of total production costs. Therefore, the breeding and cultivation of mite-resistant chestnut varieties represent the most sustainable strategy for reducing chemical inputs and fostering environmentally responsible production.

Although precise estimates remain uncertain, plants are believed to synthesize between 200,000 and one million distinct metabolites [7,8]. Variations in metabolite composition and abundance indicate plant capacity to adapt to environmental stress. The biosynthesis of secondary metabolites is a fundamental defense mechanism against both biotic and abiotic stresses [9]. In response to insect or mite attack, plants deploy both constitutive and inducible defense strategies, including growth modulation and extensive metabolic reprogramming [10,11]. Substantial progress has been achieved in characterizing these metabolic responses across a wide range of plant species. For instance, in peach, resistance to the green peach aphid (*Myzus persicae*) is associated with enhanced triterpenoid production and upregulation of genes involved in their biosynthesis [12]. In maize, the feeding by Asian corn borer (*Ostrinia furnacalis*) under field conditions stimulates defense pathways linked to phytohormones, benzoxazinoids, and volatile compounds during the mid-whorl stage [13]. Infestations by *Oligonychus coffeae* Nietner in tea and *Helopeltis theivora* Waterhouse promote the accumulation of fatty acid-derived metabolites [14], while catechins contribute to resistance against leaf miner in horse chestnut [15]. Similarly, In tea (*Camellia sinensis*), infestation by the sis-spotted spider mite (*Eotetranychus sexmaculus*) markedly enhances the upregulation of phenylpropanoid and flavonoid biosynthetic pathways, leading to pronounced accumulation of metabolites, including naringenin, quercetin, and apigenin, which actively contribute to defense against herbivory [16].

Systematic investigations of secondary metabolite accumulation patterns and temporal dynamics in Chinese chestnut under mite stress remain limited. Moreover, differences in metabolite composition and associated metabolic pathways between resistant and susceptible cultivars have not been fully elucidated. Recently, widely targeted metabolomics, primarily based on ultra-high performance liquid chromatography coupled with triple quadrupole mass spectrometry (UHPLC-QQQ-MS), has emerged as a powerful analytical platform that combines the advantages of targeted and non-targeted metabolite profiling [17]. This approach has been widely used in metabolic studies of crops and fruit trees because of its high throughput, extensive metabolite coverage, rapid chromatographic resolution, and high sensitivity [18]. Accordingly, this study offers a reliable strategy for comprehensive qualitative and quantitative assessment of secondary metabolism in Chinese chestnut tissues.

In this study, four *C. mollissima* varieties exhibiting different levels of resistance to *O. ununguis*–Yanxing (S19), Xujia 1 (R-1), Yankui (M1), and Yanshanzaofeng (M2)–were evaluated. Secondary metabolite profiles and relative abundance in leaves infested with mites were characterized using a widely targeted metabolomics platform. Comparative analyses enabled the identification of differentially accumulated metabolites (DAMs) and enriched pathways for distinguishing resistant from susceptible cultivars. This study addresses a key knowledge gap in chestnut-mite interactions and establishes a metabolomics-based framework for identifying resistant germplasm and implementing marker-assisted breeding. The findings contribute to the development of mite-resistant chestnut cultivars, thereby reducing dependence on chemical acaricides and promoting sustainable chestnut production.

## Materials and methods

### Plant materials

In 2021, four Chinese chestnut varieties (Yanxing, Xujia 1, Yankui, and Yanshanzaofeng) were cultivated at the Chestnut Germplasm Resource Nursery of the Changli Institute of Pomology, Hebei Academy of Agricultural and Forestry Sciences, Qinhuangdao, Hebei Province, China (39°45′ N, 119°12′ E). Leaf samples were collected from the middle sections of vegetative branches. All visible insects were removed before samples were immediately stored at −80°C in an ultra-low temperature freezer to preserve tissue integrity.

### Field evaluation of mite resistance in Chinese chestnut varieties

Field trials were conducted to assess mite resistance in accordance with the descriptors and data standards for *C. mollissima* [19]. Ten trees of comparable growth were selected for each cultivar and divided into five survey trees and five control trees. The survey trees were left untreated throughout the growing season, whereas the control trees were sprayed with pesticides during chestnut red mite activity to minimize infestation. In early August, chlorotic areas were quantified on 50 leaves per tree using the grading standard presented in Table 1.

**Table 1:** Grading standard for leaf chlorosis in Chinese chestnut after mite infestation.

Standard score	Description
**0**	Leaf blade nearly completely green
**1**	Chlorotic area < 25% of the total leaf area
**2**	Chlorotic area 26–50% of the total leaf area
**3**	Chlorotic area 51–75% of the total leaf area
**4**	Chlorotic area 76–100% of the total leaf area

The leaf chlorosis index (*I)* was calculated using the following formula:


$$\begin{array}{c} I=\frac{\sum (x*n)}{X*N}\times \text{100} \end{array}$$ (1)

where *x* denotes the chlorosis grade value of each leaf, *n* denotes the number of leaves at that grade, *X* represents the maximum grade value, and *N* is the total number of leaves evaluated. Chestnut resistance to red mite was categorized as follows based on the index: highly resistant (*I* < 15%), resistant (15% ≤ *I* < 25%), moderately resistant (25% ≤ *I* < 35%), sensitive (35% ≤ *I* < 60%), or highly sensitive (*I* ≥ 60%).

### Collection of leaf samples for metabolomic analysis

Leaf samples were collected concurrently with the field evaluation, based on mite resistance phenotypic data in *C. mollissima*. Each biological replicate comprised nine plants that were exposed to mite damage. From peripheral vegetative branches, 20 leaves per replicate were pooled, carefully inspected to remove any impurities (e.g., insects or debris), immediately flash-frozen in liquid nitrogen, and stored at −80°C until further analysis.

### Preparation of samples for metabolomic analysis

The freeze-dried leaf samples were ground using a mixer mill (MM 400, Retsch, Haan, Germany) at 30 Hz for 1.5 min. 100 mg of each powdered sample was extracted with 1.2 mL of 70% aqueous methanol overnight at 4°C. The extracts were then centrifuged at 12,000 × g for 10 min, and the supernatant was purified using a CNWBOND Carbon-GCB SPE Cartridge (250 mg, 3 mL; ANPEL, Shanghai, China). The purified extracts were filtered through a 0.22 μm membrane filter (SCAA-104; ANPEL, Shanghai, China) and transferred to glass vials for downstream analyses. QC samples were prepared by pooling equal volumes of extracts from the four varieties, processed using the same procedure, and used to monitor stability and reproducibility.

### Analytical setup for metabolomic profiling

Metabolomic analysis was performed using an LC-ESI-MS/MS system (High-performance liquid chromatography: Shim-pack Ultra-fast liquid chromatography, Shimadzu CBM30A; MS: Applied Biosystems 4500 Q TRAP, Framingham, MA, USA). Chromatographic separation was performed using a Waters ACQUITY UPLC HSS T3 C18 column (1.8 µm, 2.1 × 100 mm). The mobile phases consisted of 0.04% acetic acid in water (solvent A) and 0.04% acetic acid in acetonitrile (solvent B). The elution gradient was programmed as follows: 95% A and 5% B at the start, linearly shifting to 5% A and 95% B over 10 min, held for 1 min, then returned to 95% A and 5% B within 0.1 min, followed by a 2.9-min re-equilibration. The column temperature was maintained at 40°C, and the injection volume was 4 μL. Mass spectrometry was conducted in positive and negative ion modes, with ion spray voltages of 5500 V and −4500 V, respectively, and a source temperature of 550°C. Ion source gases I (GSI) and II (GSII) were set to 50 and 60 psi, curtain gas (CUR) was set to 30 psi, and collision gas (CAD) was set to high. In the QQQ mode, nitrogen was used as the CAD at 5 psi.

### Qualitative and quantitative analysis of metabolites

Metabolite identification and quantification were performed using mass spectrometry (MS) in combination with publicly available databases and the self-constructed MetWare Database (Wuhan MetWare Biotechnology Co., Ltd., Wuhan, China). Quantitative analysis was conducted in the MRM mode using a QQQ MS [17]. Metabolite-specific ion transitions were selected to produce corresponding signal intensities in MRM mode, and the resulting chromatographic peaks were processed and quantified using MultiQuant software. The integration peak areas represent the relative abundance of the corresponding metabolites.

### Evaluation of analytical precision and reproducibility

To evaluate the instrumental stability and analytical repeatability under mite stress conditions, QC samples were prepared by pooling aliquots from all sample extracts and were injected after every 10 experimental samples [20]. Analytical accuracy and reproducibility were assessed by superimposing TIC profiles of QC runs to examine signal consistency and stability of retention time.

### Multivariate and statistical analyses

Raw metabolomic data were normalized according to previously established procedures [21] and were subsequently subjected to log_2_ transformation before analysis. PCA, HCA, and OPLS-DA were conducted using the R software (http://www.r-project.org/) following standard analytical workflows.

### Differential analysis of metabolites

The OPLS-DA, a supervised multivariate approach, was used to identify DAMs between resistant and susceptible chestnut varieties. Metabolites with a variable importance in projection (VIP) ≥ 1 and fold change (FC) ≥ 2 or ≤ 0.5 were considered significantly different. Identified DAMs were annotated using the KEGG database (http://www.kegg.jp/kegg/pathway.html) [22]. Pathway enrichment analysis was performed using a hypergeometric test against the background metabolite set, with statistical significance defined as *P* < 0.05.

### RNA isolation and RNA-seq analysis

Raw sequencing reads in FASTQ format were subjected to quality filtering using fastp to eliminate adapter contamination, duplicate sequences, and low-quality reads. The filtered clean reads were subsequently mapped to the C. mollissma reference genome using HISAT2 (v 2.2.2.0), generating binary alignment map (BAM) files [23]. Transcript assembly was performed using StringTie, and gene expression abundance was estimated as FPKM (fragments per kilobase of transcript per million mapped reads).

Differential expression analysis was performed between groups using DESeq2. Genes with adjusted p-values (Benjamini-Hochberg correction) < 0.05 and |log₂(fold change)| ≥ 1 were defined as significantly differentially expressed. Functional annotation and enrichment analyses were conducted using hypergeometric testing, and KEGG pathway level to identify biologically relevant processes.

## Results

### Classification of mite resistance in chinese chestnut germplasm

**Fig 1:**
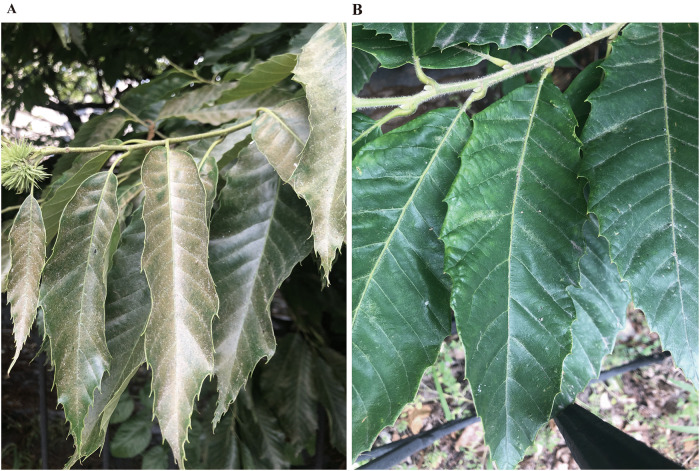
Field infestation severity in mite-susceptible and -resistant cultivars. **(A)** Susceptible cultivar; **(B)** Resistant cultivar.

Based on the leaf chlorosis index, the four germplasm accessions displayed distinct levels of mite infestation resistance. S19 was classified as highly susceptible (68.73%), whereas R-1 exhibited strong resistance (12.35%). M1 (45.38%) and M2 (37.24%) samples were also classified as susceptible. Three comparative groups were defined according to these resistance categories: R-1 versus S19, R-1 versus M1, and R-1 versus M2. These comparisons encompass both extreme (high resistance vs. high susceptibility) and intermediate (high resistance vs. susceptibility) contrasts, thereby facilitating a systematic evaluation of secondary metabolite variation and associated pathway alterations across resistance gradients. Fig 1 shows the different field infestation severity of resistant cultivar and susceptible cultivar.

### Metabolite identification and assessment of quality control

**Fig 2:**
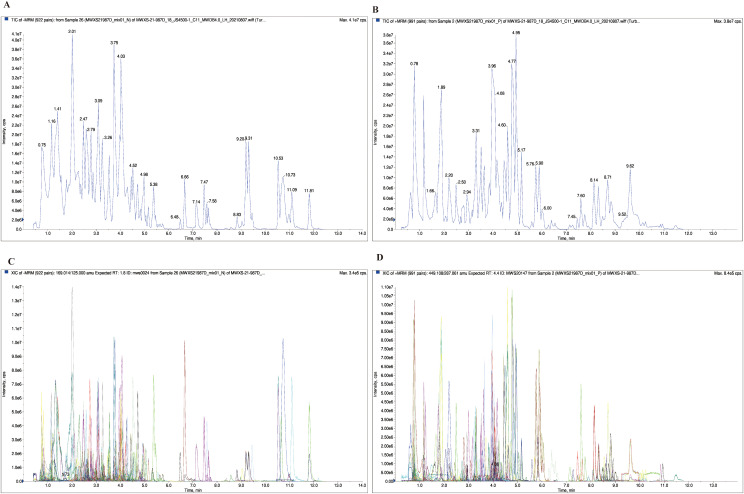
(A) Total ion current (TIC) chromatograms of QC samples in negative ion mode. **(B)** TIC chromatograms of the QC samples in the positive ion mode. **(C)** Multipeak MRM chromatograms in the negative ion mode. **(D)** Multipeak MRM chromatograms in the positive ion mode.

Fig 2 shows the total ion current (TIC) chromatograms and multi-peak detection profiles of the quality control (QC) sample. The TIC plot displays the continuous variation in summed ion intensities over the retention time, whereas the multiple reaction monitoring (MRM) multi-peak plot illustrates the simultaneous detection of diverse metabolites, with each colored peak corresponding to a specific compound. Both qualitative identification and quantitative mass spectrometric (MS) analyses were performed based on a locally constructed metabolite database. A total of 713 metabolites, including 83 flavones, 100 flavonols, 38 flavonoids, 15 flavanols, 10 isoflavones, 221 phenolic acids, 43 tannins, 58 alkaloids, 72 terpenoids, 31 lignans, 18 coumarins, one steroid, and 23 additional metabolites, were identified. Comprehensive information is provided in S1 Table in S1 File.

An overlay analysis was performed to evaluate the technical reproducibility of metabolite extraction and detection. As illustrated in Fig 2, the TIC plots of three QC samples exhibited nearly complete overlap, indicating stable instrument performance and high repeatability across analytical runs. Furthermore, the strong correlation coefficients (r = 0.989–0.998) among the three biological replicates of each sample confirmed satisfactory reproducibility and consistency (S1 Fig).

### Multivariate analysis of metabolite profiles in Chinese chestnut germplasm

**Fig 3:**
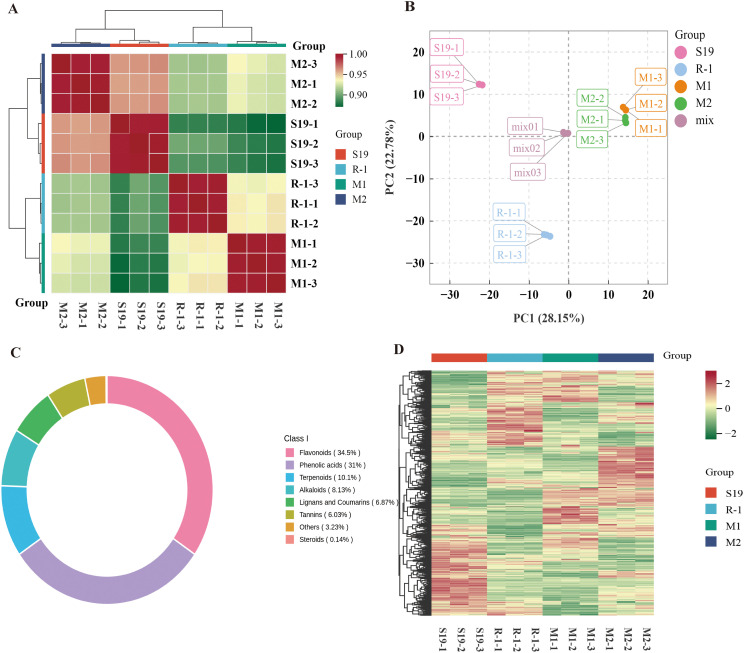
(A) Heatmap illustrating correlations among three biological replicates of four Chinese chestnut cultivars. **(B)** PCA score plot of the metabolite profiles. **(C)** Circular diagram of the detected metabolite categories. **(D)** Hierarchical clustering heatmap of the four cultivars.

A heatmap was constructed to examine correlations among biological replicates using Pearson’s correlation coefficient (PCC) as the assessment metric. Strong positive correlations were observed across all replicates (Fig 3A).

Principal component analysis (PCA) based on Liquid chromatography-electrospray ionization-tandem mass spectrometry data was obtained to evaluate the overall metabolic variation among the four chestnut germplasm accessions [24]. In the PCA score plot, PC1 and PC2 explained 28.15% and 22.78% of the total variance, respectively. M1 and M2 exhibited minimal separation, whereas R-1 was distinctly separated from the remaining samples along the PC2 axis, revealing pronounced differences in the metabolite composition between the resistant and susceptible germplasms. The close clustering of the three biological replicates for each cultivar further confirmed the robustness and reliability of the experimental data (Fig 3B).

A total of 713 secondary metabolites, including 246 flavonoids, 221 phenolic acids, 72 terpenoids, 58 alkaloids, 49 lignans and coumarins, 43 tannins, 23 miscellaneous compounds, and one steroid, were detected in the leaves of the four chestnut cultivars. Flavonoids and phenolic acids constituted the predominant categories, representing 34.5% and 31% of the total metabolite content, respectively. In contrast, steroids and other minor compounds accounted for only 0.14% and 3.23% of the total, respectively (Fig 3C).

Hierarchical cluster analysis (HCA) of these bioactive metabolites grouped the samples into four distinct clusters in the heatmap. This separation reflects substantial variation in metabolite abundance among the chestnut varieties, indicating that genetic differences markedly influence metabolic profiles associated with resistance to red mites (Fig 3D).

### Discriminant analysis of metabolite profiles in response to mite stress

**Fig 4:**
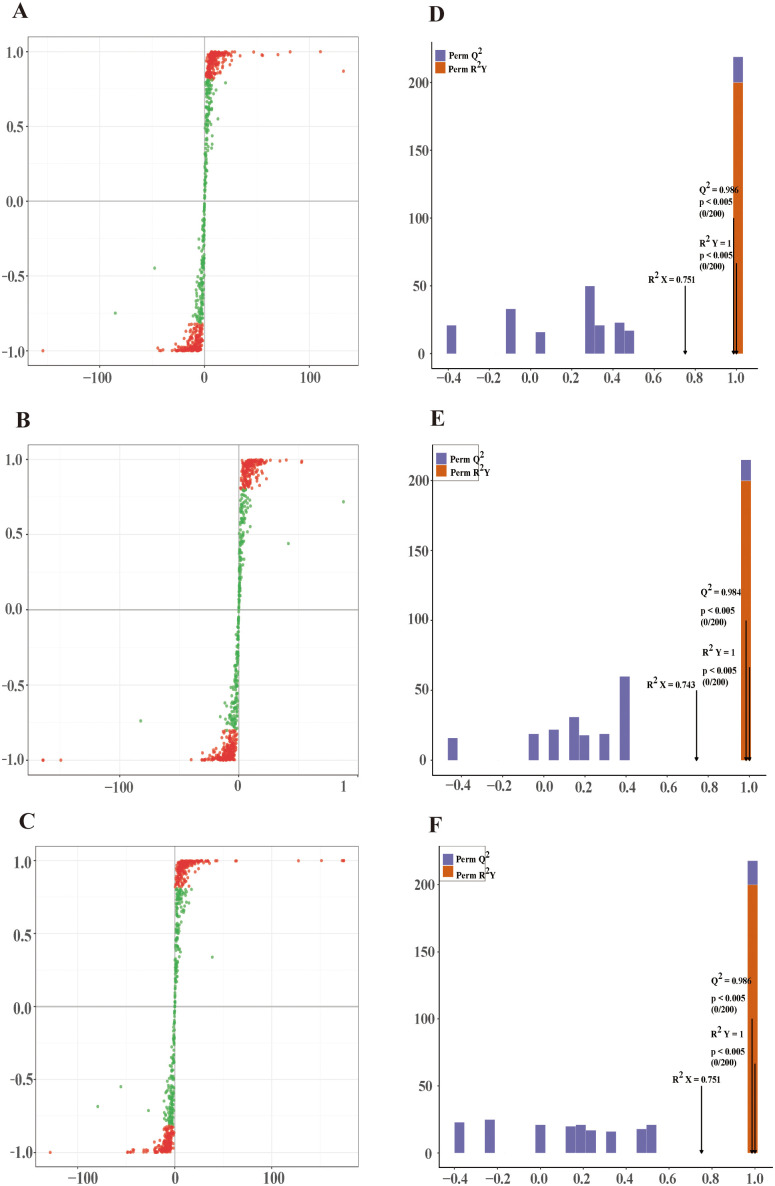
Orthogonal partial least squares-discriminant analysis (OPLS-DA) score plots of four Chinese chestnut germplasm accessions.(A, D) M1 vs. R-1; (B, E) R-1 vs. M2; (C, F) S19 vs. R-1.

Orthogonal partial least squares-discriminant analysis (OPLS-DA), a supervised multivariate approach, integrates partial least squares-discriminant analysis (PLS-DA) with orthogonal signal correction (OSC), partitioning the X matrix into variation correlated with Y and variation independent of Y [25]. Differentially responsive variables are identified by removing the orthogonal (non-correlated) components. OPLS-DA was used to discriminate metabolic differences between resistant and susceptible cultivar pairs: M1 vs. R-1 (R^2^Y = 1, R^2^X = 0.751, Q^2^ = 0.986), R-1 vs. M2 (R^2^Y = 1, R^2^X = 0.743, Q^2^ = 0.984), and S19 vs. R-1 (R^2^Y = 1, R^2^X = 0.751, Q^2^ = 0.986) (Fig 4D–4F). All models displayed R^2^X values exceeding 0.55, R^2^Y values above 0.99, and Q^2^ values greater than 0.90, indicating strong explanatory and predictive capacity. The OPLS-DA score plots demonstrated clear separation between comparison groups, reflecting distinct metabolomic responses to mite stress in resistant versus susceptible varieties. The corresponding S-plots are presented in Fig 4A–4C. Model robustness was further confirmed through 200 permutation tests to validate reliability and exclude overfitting.

### Differential metabolite discovery and pathway enrichment analysis

**Fig 5:**
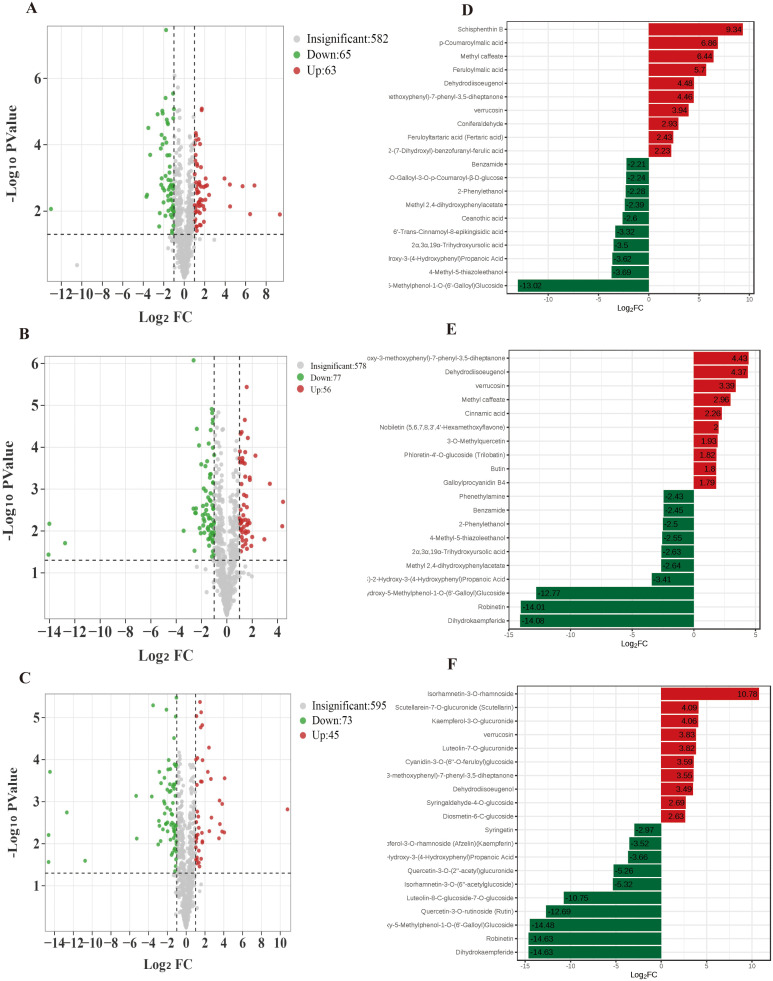
DAMS volcano plots in pairwise comparisons: (A) M1 vs. R-1; (B) R-1 vs. M2; (C) S19 vs. R-1. Bar charts of fold-change (FC) differences: **(D)** M1 vs. R-1; **(E)** R-1 vs. M2; **(F)** S19 vs. R-1. Red indicates upregulation and green indicates downregulation.

DAMs were identified in all three pairwise comparisons using a variable importance in projection (VIP) threshold ≥ 1 combined with a fold change (FC) criteria of ≤ 0.5 or ≥ 2. A total of 128 DAMs were detected in M1 vs. R-1 (63 upregulated, 65 downregulated), 133 in R-1 vs. M2 (56 upregulated, 77 downregulated), and 118 in S19 vs. R-1 (45 upregulated, 73 downregulated). Volcano plots depicting these differences are presented in Fig 5A–5C, and the top-ranked metabolites based on log_2_FC are shown in Fig 5D–5F.

**Fig 6:**
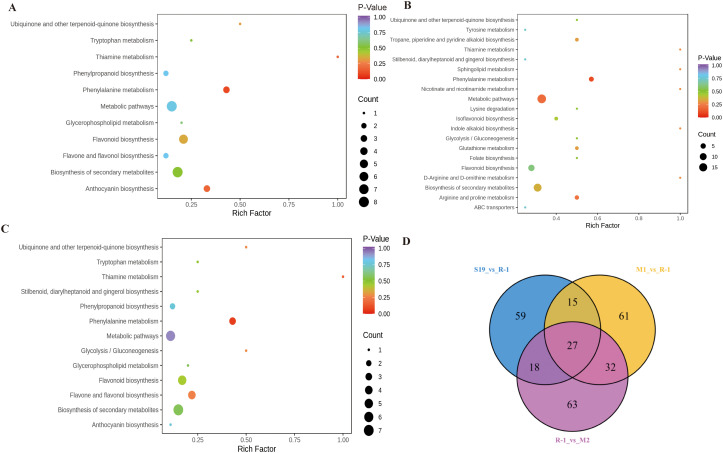
KEGG enrichment maps of DAMs across pairwise comparisons: (A) M1 vs. R-1; (B) R-1 vs. M2; (C) S19 vs. R-1. **(D)** Venn diagram illustrating the shared and unique DAMs across the three comparisons.

All dams were subsequently mapped to the KEGG database, a centralized resource linking gene expression to metabolite accumulation and metabolic pathways. Pathway enrichment analysis categorized DAMs into specific biochemical pathways. Comparisons of the resistant cultivar R-1 with the susceptible cultivars M1, M2, and S19 revealed enrichment in pathways 14, 20, and 13, respectively. The major pathways for each comparison are illustrated in the KEGG bubble plots (Fig 6).

Differentially accumulated metabolites were substantially enriched in phenylalanine metabolism, as well as flavonoid and anthocyanin biosynthetic pathways, between M1 and R-1. Flavonoid biosynthesis and general secondary metabolite biosynthesis accounted for the largest number of DAMs, highlighting their pivotal role in distinguishing the two cultivars’ metabolic profiles. In R-1 vs. M2, phenylalanine metabolism, broad metabolic pathways, and flavonoid biosynthesis metabolites were enriched, with additional compounds mapped to secondary metabolite production as well as arginine and proline metabolism. Similarly, significant enrichment was detected in phenylalanine metabolism and flavonoid biosynthesis in S19 vs. R-1, with numerous metabolites also assigned to general metabolic pathways, flavonoid biosynthesis, and secondary metabolite formation.

A total of 275 DAMs were identified among all three comparisons between R-1 and the susceptible cultivars (S19, M1, and M2). The Venn diagram analysis revealed 27 shared metabolites among these comparisons (Table 2 and Fig 6D). These metabolites were classified into eight bioactive categories: steroids, terpenoids, alkaloids, tannins, lignans and coumarins, flavonoids, phenolic acids, and other compounds (Table 3). The 27 common metabolites, including phenolic acids, flavonoids, alkaloids, and terpenoids exhibited consistent accumulation patterns across all groups. In the resistant cultivar, six compounds (cinnamic acid, methyl caffeate, baicalein, gallocatechin, dehydrodiisoeugenol, and lucidenic acid) were specifically accumulated at elevated levels. Among the 27 shared metabolites, 15 displayed highly significant differences (*P* < 0.01), with 11 downregulated and 4 upregulated. KEGG pathway annotation indicated that five metabolites (2-phenylethylamine, 2-phenylethanol, 4-methyl-5-thiazolylethanol, cinnamic acid, and gallocatechin) were predominantly associated with stress-responsive pathways, including flavonoid metabolism (ko00941), phenylalanine metabolism (ko00360), and phenylpropanoid biosynthesis (ko00940).

**Table 2:** Twenty-seven common DAMs across all comparison groups.

No.	Metabolite	Significance	Class	Regulation		
				S19 vs. R-1	M1 vs. R-1	M2 vs. R-1
**1**	Benzamide	**	Phenolics	down	down	down
**2**	Phenethylamine	**	Alkaloids	down	down	down
**3**	2-Phenylethanol	**	Phenolics	down	down	down
**4**	4-Methyl-5-thiazoleethanol	*	Others	down	down	down
**5**	Cinnamic acid	**	Phenolics	up	up	up
**6**	Methyl 2,4-dihydroxyphenylacetate	**	Phenolics	down	down	down
**7**	(S)-2-Hydroxy-3-(4-hydroxyphenyl) propanoic acid	**	Phenolics	down	down	down
**8**	Methyl caffeate	*	Phenolics	up	up	up
**9**	Baicalein	*	Flavones	up	up	up
**10**	Gallocatechin	**	Flavanols	up	up	up
**11**	Dehydrodiisoeugenol	**	Lignans	up	up	up
**12**	1-(4-hydroxy-3-methoxyphenyl)-7-phenyl-3,5-diheptanone	**	Others	down	down	down
**13**	O-Feruloyl 4-hydroxycoumarin	*	Coumarins	down	down	down
**14**	Verrucosin	**	Lignans	up	up	up
**15**	3-Hydroxy-5-methylphenol-1-O-(6’-galloyl) glucoside	*	Phenolics	down	down	down
**16**	Ginnalin A (2,6-Di-O-Galloyl-1,5-anhydro-D-glucitol)	*	Phenolics	down	down	down
**17**	Maplexin D (2,4-Di-O-galloyl-1,5-anhydro-D-glucitol)	**	Phenolics	down	down	down
**18**	Ceanothic acid	**	Triterpenes	down	down	down
**19**	2α,19α-Dihydroxy-3-oxours-12-en-28-oic acid	**	Triterpenes	down	down	down
**20**	1,6-Di-O-caffeoyl-β-D-glucose	*	Phenolics	down	down	down
**21**	3,6-Di-O-caffeoyl glucose	**	Phenolics	down	down	down
**22**	2α,3α,19α-Trihydroxyursolic acid	**	Triterpenes	down	down	down
**23**	3-Hydroxy-5-Methylphenol-1-O-(6-digalloyl) glucoside	*	Phenolics	down	down	down
**24**	Phloretin	*	Chalcones	up	up	up
**25**	Kaempferol-3-O-rhamnoside (Afzelin) (Kaempferin)	*	Flavonols	down	up	down
**26**	Kaempferol-3-O-(4''-O-p-coumaroyl) rhamnoside	*	Flavonols	down	down	up
**27**	Cyanidin-3-O-(6''-O-feruloyl) glucoside	*	Anthocyanidins	up	down	down

Upregulation and downregulation indicate accumulation in the susceptible group compared with the resistant group. Significance levels: *P* < 0.05 (*), *P* < 0.01 (**).

**Table 3:** Differentially accumulated metabolites in *Castanea mollissima* leaves across cultivars with varying levels of resistance to *Oligonychus ununguis.*

Class	S19 vs. R-1		M1 vs. R-1		M2 vs. R-1	
	Up	Down	Up	Down	Up	Down
**Others**	1	1	3	2	3	3
**Steroids**	0	1	0	0	0	1
**Terpenoids**	1	4	1	19	4	17
**Alkaloids**	4	1	7	3	1	10
**Tannins**	0	4	7	1	8	1
**Lignans and coumarins**	10	4	6	3	5	6
**Flavonoids**	19	29	23	17	29	14
**Phenolic acids**	10	30	18	25	11	27

Upregulation and downregulation indicate relative accumulation in susceptible compared to resistant cultivar.

### Gene expression changes in different resistance groups

**Fig 7:**
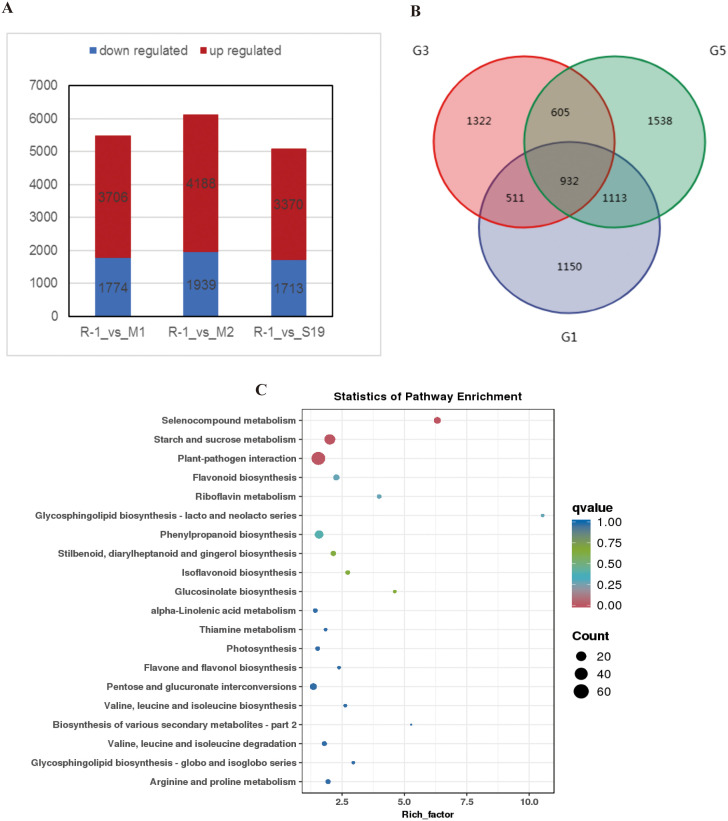
RNA-seq analyses of four chestnut leaf samples: (A) Number of DEGs identified in three pairwise comparisons. **(B)** Venn diagram showing shared DEGs among M1 vs. R-1 (G1), S19 vs. R-1 (G3), and R-1 vs. M2 (G5). **(C)** KEGG pathway enrichment of shared DEGs in G1, G3, and G5.

Ribonucleic acid (RNA) was performed on the four chestnut leaf samples to characterize transcriptional responses to mite infestation. All sequencing libraries generated high-quality reads, with Q20 and Q30 scores exceeding 99.54% and 97.91%, respectively. Differential expression analysis identified 5,480, 6,127, and 5,083 DEGs in the comparisons of R-1 vs. M1, R-1 vs. M2, and R-1 vs. S19, respectively, with upregulated genes consistently outnumbering downregulated genes in all cases (Fig 7A). A total of 932 DEGs were found to be common across all three pairwise comparisons (Fig 7B). KEGG pathway enrichment of these shared DEGs revealed consistent involvement in selenocompound metabolism (ko00450), starch and sucrose metabolism (ko00500), and plant-pathogen interaction (ko04626) (Fig 7C).

### Integrated transcriptomic and metabolomic analysis

**Fig 8:**
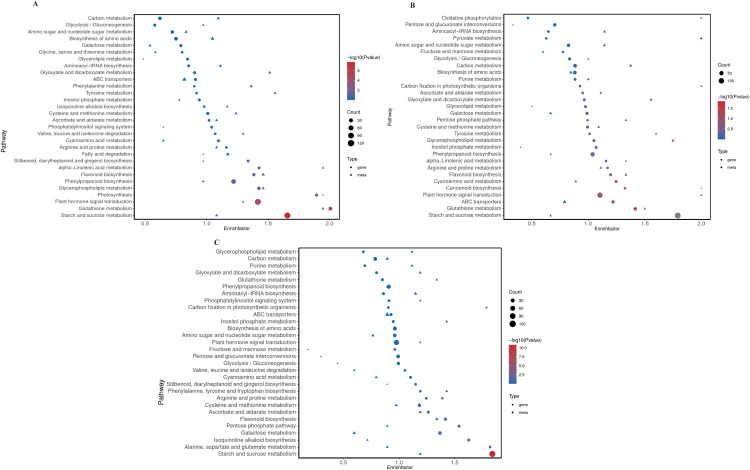
KEGG enrichment bubble plots of the top 30 pathways with significant enrichment of differentially expressed genes and metabolites: (A) M1 vs. R-1; (B) R-1 vs. M2; (C) S19 vs. R-1.

In the M1 vs. R-1 comparison, the most prominently enriched pathways among DEGs were starch and sucrose metabolism (ko00500), plant hormone signal transduction (ko4075), and glutathione metabolism (ko00480). For S19 vs. R-1 starch and sucrose metabolism (ko00500) emerged as the primary DEG-enriched pathway. In M2 vs. R-1, DEGs were chiefly enriched in glutathione metabolism (ko00480), while differentially expressed metabolites were predominantly associated with pyruvate metabolism (ko00620) (Fig 8). Collectively, these findings indicate that the defensive mechanisms of Chinese chestnut against mite infestation constitute a complex, multilayered network, involving coordinated modulation of signal transduction, energy metabolism, and innate immune responses, among other physiological and biochemical processes.

## Discussion

As an economically important forest species, Chinese chestnut production is continually challenged by red spider mite (*O. ununguis*) infestation. Substantial variation in resistance to this pest has been documented among various cultivars. However, progress in identifying resistance-related germplasm resources remains limited, and the molecular and metabolic bases of chestnut defense against mite attack are still insufficiently defined. This deficiency has hindered the advancement of molecular breeding strategies aimed at enhancing mite resistance. The present study employed an integrated transcriptomic and metabolomic approach to examine four chestnut germplasm accessions exhibiting contrasting phenotypes under *O. ununguis* stress to clarify key regulatory pathways and identifying metabolites associated with resistance.

Metabolomics provides a robust platform for investigating plant growth, adaptation, and defense under adverse conditions and has been extensively used to explore plant responses to biotic stressors. However, the metabolic defense mechanisms of Chinese chestnut (*C. mollissima*) against *O. ununguis* infestation remain poorly characterized. In this study, 71 DAMs were predominantly enriched in the flavone/flavonol and phenylpropanoid biosynthetic pathways. The phenylpropanoid pathway contributes to cell wall strengthening via lignin formation [26,27], and enhanced secondary wall deposition is a critical factor in resistance to both biotic and abiotic stress [28–34]. Flavone and flavonol biosynthesis also functions as a central node in biotic and abiotic stress as well as secondary metabolism [35,36], generating bioactive compounds such as quercetin and anthocyanins with antioxidant, antimicrobial, and indirect defense roles, including the attraction of natural enemies. Following mite herbivory, chestnut trees rapidly elevate stress-induced secondary metabolites, thereby increasing the number of defensive compounds that that can deter herbivores or recruit natural predators [37]. Comparable inducible responses have been reported in *Castanea henryi* during chestnut gall wasp infestation, where alterations in tannin content were closely associated with enhanced resistance [38].

In this study, 27 DAMs were identified through the Venn diagram analysis. Several compounds, including cinnamic acid, methyl caffeate, baicalein, gallocatechin, dehydrodiisoeugenol, and lucolipin exhibited markedly higher accumulation in the resistant genotype, indicating their potential contribution to chestnut resistance against the red spider mite. Cinnamic acid, a pivotal intermediate of the phenylpropanoid pathway, participates in cell wall lignification and reinforces tissue barriers; it has also been associated with soybean resistance to *Cotinomyces nuclealis* and demonstrated repellent activity against poplar longhorn beetles [39,40]. Baicalein exhibits anti-inflammatory, antioxidant, and antiviral properties, and its ability to suppress digestive enzymes highlights its potential as a botanical insecticide/acaricide [41,42]. Gallocatechin, a precursor of condensed tannins, plays a crucial role in chemical defense by impairing insect feeding behavior, digestion, and metabolic processes [36]. Dehydrodiisoeugenol, which is involved in lignin biosynthesis, contributes to cell wall strengthening and may additionally disrupt insect digestive enzymes or trigger oxidative stress in pathogens [43]. Luciferin, a triterpenoid, inhibits direct insect feeding [44], mitigates reactive oxygen species (ROS), and stimulates the expression of defense-related genes. The coordinated biosynthesis and regulation of these metabolites illustrate the evolution of a multilayered defense architecture in plants over time and provide a conceptual foundation for environmentally friendly pesticide innovation and the genetic improvement of stress-resistant fruit tree cultivars.

This study demonstrated that the mite-resistant cultivar exhibited a greater number of DEGs following infestation, indicating a robust capacity for signal perception and downstream transduction. Basal plant immunity is initiated through the recognition of conserved molecular patterns associated with microbes or damage-associated molecular patterns [45,46]. Infestation of *O. ununguis* activated extensive signaling cascades in Chinese chestnut, consistent with the canonical mechanisms by which plants respond to biotic stress. The integrated analysis of DEGS and DEMs further identified core pathways co-enriched in starch and sucrose metabolism, plant hormone signal transduction, and glutathione metabolism. These findings suggest that mite-infestation challenge imposes substantial energetic demands to sustain defense responses. The tight coordination between signaling pathways and energy metabolism underscores the integrated regulation of primary and defense metabolism under stress conditions [47].

Plant responses to spider mite attack involve intricate reprogramming, including specialized metabolite biosynthesis [48,49] and hormone signaling network modulation [50]. This study systematically elucidated the multilayered defense network of Chinese chestnut in response to *O. ununguis* infestation and clarified the central roles of the flavonoid and phenylpropanoid biosynthetic pathways in anti-mite defense through combined transcriptomic and metabolomic analyses. Several candidate resistance-associated metabolites, including cinnamic acid, methyl caffeate, baicalin, gallocatechin, dehydrodiisoeugenol, and chlorohexin, specifically accumulated in highly resistant germplasm, supporting their potential contributions to mite resistance. This study addresses a critical gap in understanding the interaction between chestnut and mites, offering novel insights into the molecular basis of resistance in woody plants to piercing-sucking arthropods such as *O. ununguis*. However, several limitations should be acknowledged. First, the conclusions are primarily derived from integrative omics-based correlation analyses, and genetic transformation or biochemical assays have not directly validated the roles of key candidate genes and metabolites. Second, the investigation centered on leaf tissues sampled at specific time points, which may not fully capture the anti-mite defense’s temporal dynamics. Future research should therefore prioritize functional validation of candidate transcription factors and structural genes using transgenic approaches or genome-editing technologies, as well as validation experiments such as exogenous application (e.g., spraying) of candidate metabolites to confirm their direct role in enhancing plant resistance. In addition, time-course analyses across multiple cultivars should be conducted to elucidate dynamic defense trajectories and to establish comprehensive resistance evaluation indices. Such efforts would provide theoretical and practical foundation for molecular design breeding and precision-based ecological management strategies.

## Conclusion

This study presents the first comprehensive characterization of a multilayered and coordinated defense network in Chinese chestnut in response to infestation to *O. ununguis* infestation using integrated transcriptomic and metabolomic approaches. The results highlight the central contributions of the flavonoid and phenylpropanoid biosynthetic pathways to mite resistance and identify several resistance-associated metabolites that preferentially accumulate in highly resistant germplasms, including cinnamic acid, methyl caffeate, baicalin, gallocatechin, dehydrodiisoeugenol, and chlorolipid, indicating their potential functional roles in defense. These findings address a significant a significant knowledge gap in chestnut-mite interactions and advance the current understanding of the molecular mechanisms underlying woody plant resistance to piercing-sucking arthropods.

## Supporting information

S1 File S1 Fig. Reproducibility of biological replicates. The strong correlation coefficients (r = 0.989–0.998) among the three biological replicates of each sample confirmed satisfactory reproducibility and consistency. S1 Table. Overview of identified metabolite classes. A total of 713 metabolites were identified, comprising 83 flavones, 100 flavonols, 38 flavonoids, 15 flavanols, 10 isoflavones, 221 phenolic acids, 43 tannins, 58 alkaloids, 72 terpenoids, 31 lignans, 18 coumarins, one steroid, and 23 additional metabolites. Detailed information is provided in this table.
(ZIP)
